# Correction: Charged metabolite biomarkers of food intake assessed via plasma metabolomics in a population-based observational study in Japan

**DOI:** 10.1371/journal.pone.0250864

**Published:** 2021-04-22

**Authors:** Eriko Shibutami, Ryota Ishii, Sei Harada, Ayako Kurihara, Kazuyo Kuwabara, Suzuka Kato, Miho Iida, Miki Akiyama, Daisuke Sugiyama, Akiyoshi Hirayama, Asako Sato, Kaori Amano, Masahiro Sugimoto, Tomoyoshi Soga, Masaru Tomita, Toru Takebayashi

In Table 2, the mean (10^th^-90^th^ range)^a^ of Rice for Male should be (250–680). Please see the correct Table 2 here.

**Table 2:** Food classification and population intake status.

Food group	Food item on FFQ	Mean (10^th^-90^th^ range)^a^						
		All			Male		Female	
		n = 7,012			n = 3,198		n = 3,814	
Energy-giving foods								
Rice	Rice	g/d	394	(188-600)	485	(250-680)	317	(165-450)
Other grains/potatoes	Bread, noodles, soba, potatoes	g/d	129	(72-204)	131	(68-215)	127	(72-195)
Confectionery	Cake, Japanese traditional sweets	g/d	21	(7-42)	18	(7-28)	24	(10-47)
Oil	Butter, margarine, mayonnaise, oil for deep fried/stir fried	g/d	14	(6-24)	12	(5-22)	15	(6-25)
Protein-rich foods								
Meat	Beef/pork, chicken, liver, ham/sausage	g/d	41	(17-69)	39	(17-69)	42	(17-70)
Fish/seafood	Fish, shellfish, squid/shrimp/crab/octopus, fish roe, processed fish food, canned tuna	g/d	62	(28-98)	62	(28-100)	62	(27-97)
Eggs	Eggs	g/d	19	(4-40)	18	(4-40)	19	(8-40)
Dairy products	Milk, yogurt	g/d	122	(13-255)	99	(13-210)	142	(26-255)
Soy products	Soybeans, tofu, fermented soy food, fried soy product	g/d	112	(41-195)	111	(41-195)	113	(42-194)
Fruits/vegetables								
Carotenoid-rich vegetables	Pumpkin, carrot, broccoli, green leafy vegetables, other carotenoid-rich vegetables	g/d	78	(27-146)	63	(22-116)	92	(34-166)
Other vegetables	Cabbage, Japanese radish, dried radish, burdock, other light vegetables, mushroom	g/d	78	(28-140)	61	(24-111)	93	(35-157)
Seaweed	Seaweed	g/d	2	(1-4)	2	(1-4)	2	(1-5)
Fruits	Mandarin/orange/grapefruit, other fruits	g/d	55	(13-125)	41	(13-89)	66	(17-136)
Seeds	Peanuts/almond	g/d	3	(1-4)	3	(1-4)	3	(1-4)
Beverages								
Green tea	Green tea	g/d	230	(11-600)	220	(11-660)	239	(10-600)
Coffee	Coffee	g/d	146	(10-300)	134	(10-300)	156	(10-300)
Alcohol^b^	Sake, beer, whiskey, wine, shochu, chuhai	g/d	106	(0-377)	199	(0-480)	29	(0-93)

FFQ, food frequency questionnaire.

^a^ Values are presented as mean and 10^th^-90^th^ percentiles in parentheses.

^b^ Values are calculated according to the percentage of ethanol and shown in comparison to sake.

In Table 3, the Q^2^cum^c^ for Eggs should be (0.02). Please see the correct Table 3 here.

**Table 3:** Promising food biomarker candidates (n = 7,012).

Food group	Metabolite	Sub Class^a^	PLS-R^b^			*r*_*s*_^d^
			VIP	Coeff	*Q*^2^_cum_^c^	
Meat						
	Hydroxyproline	AA	2.66	0.07	0.07	0.09
	3-Methylhistidine	AA	2.11	0.06		0.08
	beta-Alanine	AA	2.05	0.05		0.04
	2-Aminobutyrate	AA	2.01	0.05		0.05
	Creatine	AA	1.99	0.06		0.05
	Carnitine	AA	1.70	0.04		0.03
Fish/seafood						
	Creatine	AA	3.19	0.10	0.21	0.18
	Trimethylamine-*N*-oxide	AO	2.63	0.09		0.15
	Cystine	AA	2.26	0.07		0.12
	2-Hydroxybutyrate	AA	1.73	0.04		0.11
	Isethionate	AHA	1.55	0.03		0.08
	Glucuronate	CHO	1.43	0.04		0.13
	2-Aminobutyrate	AA	1.36	0.03		0.07
	Uridine	PN	1.32	0.03		0.06
	Guanidinosuccinate	AA	1.21	0.02		0.07
Eggs						
	Choline	QA	2.88	0.05	0.01 (0.02)	0.06
	2-Aminobutyrate	AA	2.40	0.04		0.04
	Betaine	AA	2.14	0.04		0.05
	Asparagine	AA	1.66	0.02		0.02
Dairy						
	Galactarate	CHO	2.14	0.08	0.33	0.09
	Threonate	CHO	1.97	0.07		0.09
	Phenylalanine	AA	1.95	0.08		0.08
	Lysine	AA	1.60	0.04		0.05
	Tyrosine	AA	1.53	0.04		0.02
	Citrate	TCA	1.47	0.07		0.07
	Tryptophan	AA	1.44	0.02		0.03
	2-Aminobutyrate	AA	1.31	0.05		0.07
	Hippurate	BA	1.27	0.05		0.08
	Creatine	AA	1.24	0.03		0.02
Soy products						
	Cystine	AA	1.73	0.07	0.23	0.08
	Betaine	AA	1.53	0.06		0.07
	Isethionate	TCA	1.34	0.02		0.09
	Creatine	AA	1.34	0.05		0.08
	Uridine	PN	1.30	0.04		0.06
	Citrate	AA	1.25	0.04		0.06
	Phenylalanine	AA	1.25	0.03		-0.02
	Glutamine	AA	1.25	0.04		0.05
Carotenoid-rich vegetables						
	Threonate	CHO	2.23	0.07	0.28	0.09
	Galactarate	CHO	2.06	0.06		0.07
	Creatine	AA	1.80	0.06		0.05
	Lysine	AA	1.44	0.02		0.03
	Cystine	AA	1.40	0.04		0.07
	Citrate	TCA	1.33	0.04		0.06
	Hippurate	BA	1.29	0.04		0.07
Other vegetables						
	Creatine	AA	2.00	0.07	0.31	0.05
	Threonate	CH	1.85	0.05		0.06
	Galactarate	CH	1.51	0.04		0.02
	Cystine	AA	1.40	0.04		0.06
Fruits						
	Proline betaine	AA	3.80	0.23	0.47	0.27
	Threonate	CHO	2.30	0.09		0.15
	Galactarate	CHO	1.95	0.07		0.11
	Tyrosine	AA	1.49	0.03		0.00
	Lysine	AA	1.43	0.02		0.03
	Cystine	AA	1.29	0.04		0.06
	Creatine	AA	1.29	0.06		0.04
	Citrate	TCA	1.21	0.05		0.06
Green tea						
	Threonate	CHO	3.54	0.06	0.05	0.11
	Galactarate	CHO	3.15	0.06		0.08
	Cystine	AA	1.93	0.04		0.07
	Creatine	AA	1.87	0.03		0.06
	2-Aminobutyrate	AA	1.74	0.03		0.06
	Trimethylamine-*N*-oxide	AO	1.71	0.03		0.07
	Proline betaine	AA	1.68	0.03		0.05
	2-Hydroxybutyrate	AA	1.29	0.02		0.06
Coffee						
	Quinate	ALC	4.59	0.29	0.55	0.39
	Trigonelline	AL	3.13	0.17		0.28
	Hippurate	BA	1.88	0.07		0.17
	Leucine	AA	1.34	0.02		0.01
Alcohol^e^						
	Pipecolate	AA	2.78	0.17	0.53	0.26
	2-Aminobutyrate	AA	1.92	0.12		0.17
	Choline	QA	1.87	0.09		0.15
	Threonine	AA	1.65	0.09		0.10
	Carnitine	AA	1.41	0.07		0.09
	Tyrosine	AA	1.34	0.06		0.08
	Malate	BHA	1.30	0.08		0.14
	Creatine	AA	1.24	0.04		0.09

PLS-R, partial least square regression; VIP, variable importance in projection; AA, amino acids, peptides, and analogs; CHO, carbohydrates and carbohydrate conjugates; AO, aminoxides; AHA, alpha-hydroxy acids and derivatives; PN, pyrimidine nucleosides; QA, quaternary ammonium salts; TCA, tricarboxylic acids and derivatives; BA, benzoic acids and derivatives; ALC, alcohols and polyols, and polyols; BHA, beta-hydroxy acids and derivatives.

^a^ Reference: The Human Metabolome Database (https://hmdb.ca)

^b^ Metabolites which indicate VIP scores ≥ 1.2 and positive PLS coefficients ≥ 0.02 are shown.

^c^ Cumulative predicted variation in the Y matrix for optimal factor numbers, calculated as 1 –(the cumulative predicted residual sum of squares / the cumulative sum of squares). The value indicates the predictive performance of the model. For cases with an optimal factor number of less than two, the factor number was set to two and the result was shown in parentheses.

^d^ Partial rank-order Spearman’s correlation coefficients between food consumption and metabolite concentration, controlling for sex, smoking, and physical activity levels.

^e^ Data of male drinkers (n = 2,449) were used in the analysis.
