# Supplementary figures and images for: Charged metabolite biomarkers of food intake assessed via plasma metabolomics in a population-based observational study in Japan
Source: PLoS One. 2021 Feb 10;16(2):e0246456. doi: 10.1371/journal.pone.0246456 (PMC7875413; doi:10.1371/journal.pone.0246456)

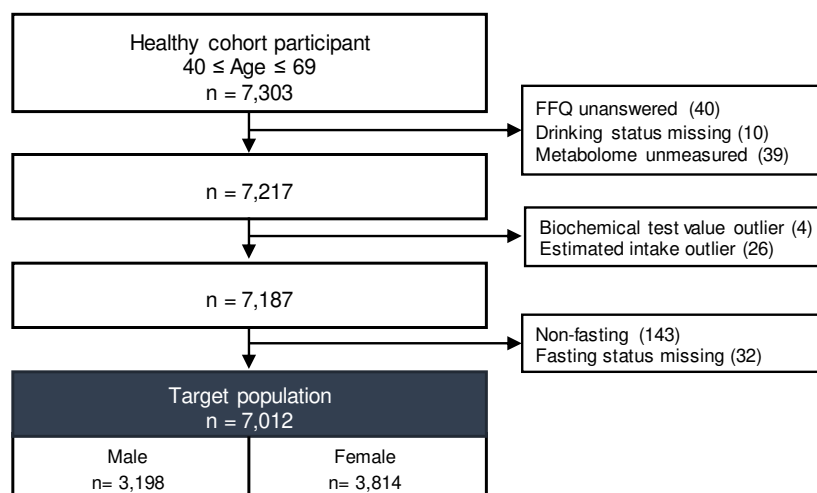

**S1 Fig. Flow diagram of participant inclusion and exclusion in the present study**

Supplement: S1 Fig — (PDF) [file pone.0246456.s001.pdf]
