# Supplementary material for: Charged metabolite biomarkers of food intake assessed via plasma metabolomics in a population-based observational study in Japan
Source: PLoS One. 2021 Feb 10;16(2):e0246456. doi: 10.1371/journal.pone.0246456 (PMC7875413; doi:10.1371/journal.pone.0246456)
Supplement: S2 Fig — Principal component (PC) analysis was executed for detecting outliers and excluded two samples from the analysis beforehand. (A) 3-D score plots for PC1, PC2, and PC3, (B) 2-D scatter plots of scores and loadings for PC1, PC2, and PC3, and (C) T2 statistics for 12 principal components. UCL, upper control limit. (PDF) [file pone.0246456.s002.pdf]

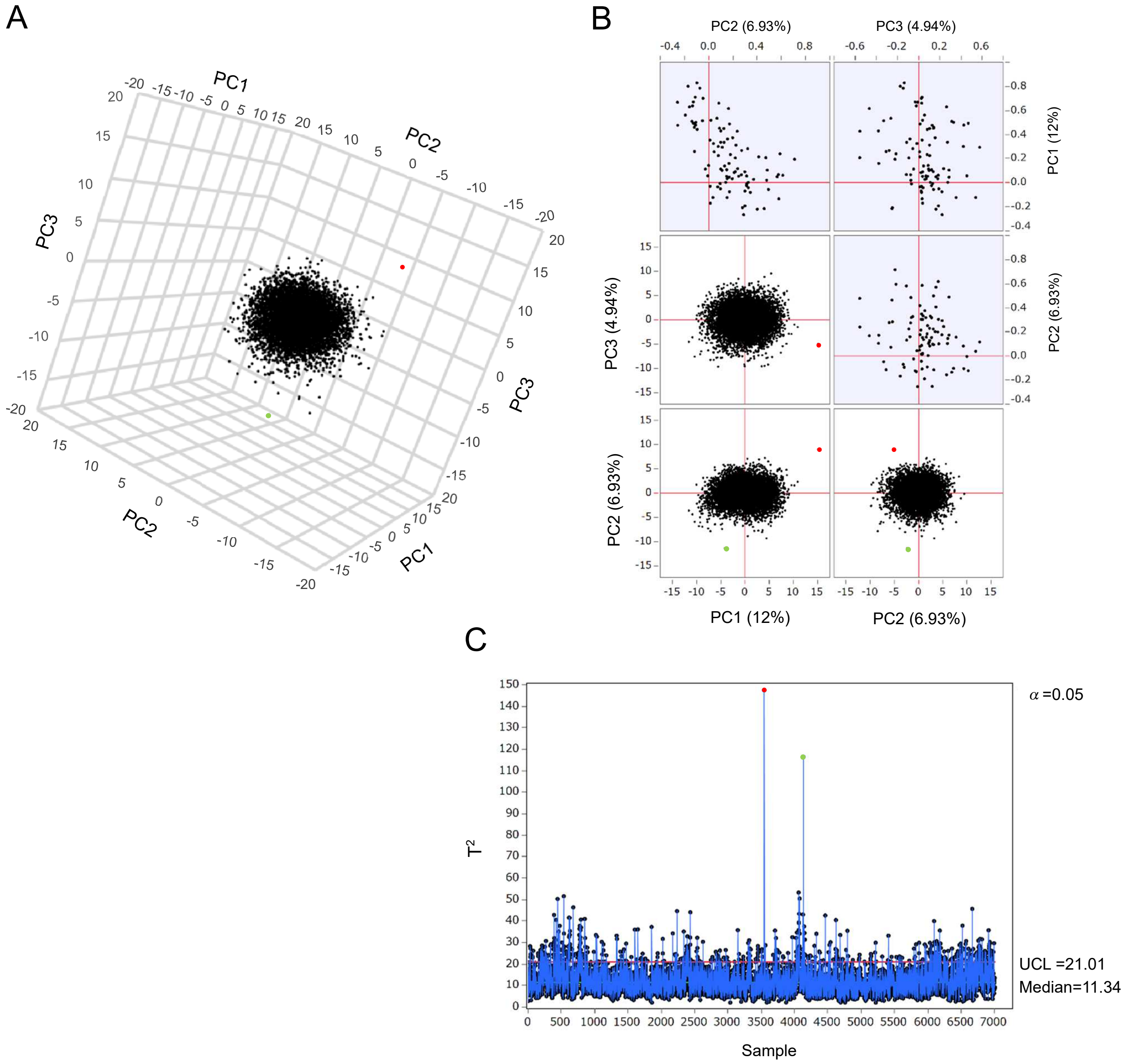

S2 Fig. Outlier detection analysis. Principal component (PC) analysis was executed for detecting outliers and excluded two samples from the analysis beforehand. (A) 3-D score plots for PC1, PC2 and PC3, (B) 2-D scatter plots of scores and loadings for PC1, PC2 and PC3, and (C) T^2 for 12 principal components. UCL, upper control limit
