# Supplementary material for: Charged metabolite biomarkers of food intake assessed via plasma metabolomics in a population-based observational study in Japan
Source: PLoS One. 2021 Feb 10;16(2):e0246456. doi: 10.1371/journal.pone.0246456 (PMC7875413; doi:10.1371/journal.pone.0246456)
Supplement: S3 Fig — As we applied Van der Voet’s test to avoid over-fitting, the number of factors extracted was the lowest with residuals that were insignificantly larger than the residuals of the model with the minimum predicted residual sum of squares (PRESS). (A) meat, (B) fish/seafood, (C) eggs, (D) dairy, (E) soy products, (F) carotenoid-rich vegetables, (G) other vegetables, (H) fruits, (I) coffee, (J) green tea, and (K) alcohol. (PDF) [file pone.0246456.s003.pdf]

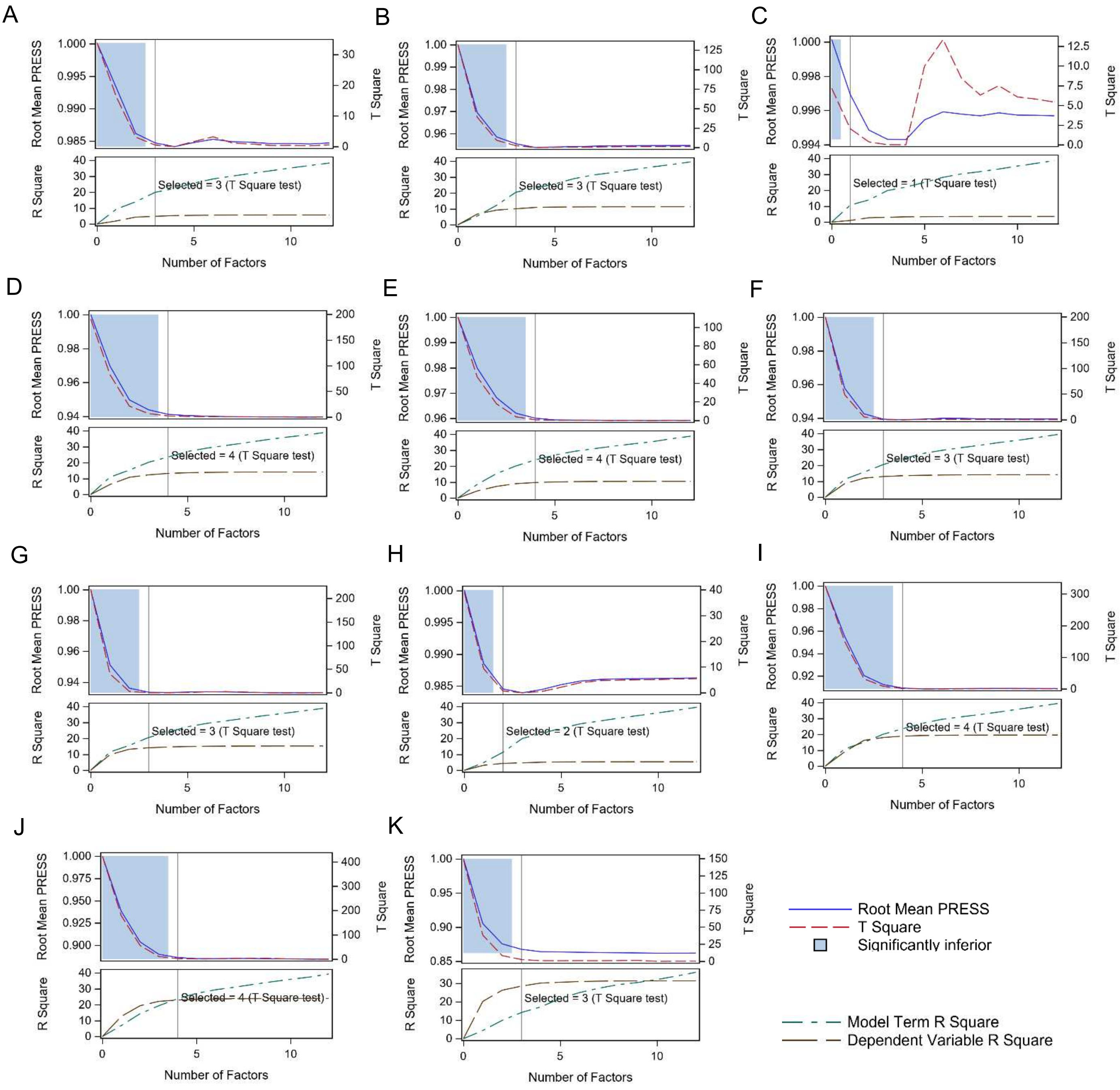

S3 Fig. Cross-validation analyses for the goodness-of-fit of models. As we applied Van der Voet tes to avoid over-fitting, the number of factors extracted was the lowest with residuals that were insignificantly larger than the residuals of the model with the minimum predicted residual sum of squares (PRESS). (A) meat, (B) fish/seafood, (C) eggs, (D) dairy, (E) soy products, (F) carotenoid-rich vegetables, (G) other vegetables, (H) fruits, (I) coffee, (J) green tea, and (K) alcohol
