# Supplementary material for: Charged metabolite biomarkers of food intake assessed via plasma metabolomics in a population-based observational study in Japan
Source: PLoS One. 2021 Feb 10;16(2):e0246456. doi: 10.1371/journal.pone.0246456 (PMC7875413; doi:10.1371/journal.pone.0246456)
Supplement: S1 Table — (PDF) [file pone.0246456.s004.pdf]

S1 Table. Questionnaire contents of the FFQ and the Lifestyle Questionnaire

A. List of food included in the questionnaires

Staple food	
	Rice
	Bread (e.g. white bread, pastry)
	Noodles (wheat e.g. Japanese noodle, Chinese noodle)
	Soba (buckwheat noodle)
**Food item**	
1	Margarine
2	Butter
3	Milk
4	Yogurt
5	Miso soup
6	Tofu (cold/boiled)
7	Natto (fermented soy food), soybean
8	Egg
9	Chicken
10	Beef, Pork
11	Liver
12	Ham, sausage, bacon, salami-sausage
13	Fish
14	Bone-edible small fish
15	Canned tuna
16	Squid, octopus, shrimp, crab
17	Shellfish (e.g. clam, oyster)
18	Fish roe (e.g. cod fish roe, salted salmon roe)
19	Processed fish food (e.g. kamaboko, chikuwa)
20	Fried soy product (e.g. ganmodoki, astu-age)
21	Potatoes (e.g. potato, sweet-potato, taro)
22	Pumpkin
23	Carrot
24	Broccoli
25	Green leafy vegetables (e.g. spinach, komatsuna)
26	Other carotenoid-rich vegetables (e.g. green pepper)
27	Cabbage
28	Japanese radish
29	Dried radish
30	Burdock, bamboo shoot
31	Other light vegetables (e.g. cucumber, onion, lettuce)
32	Mushrooms (e.g. shiitake, shimeji, enoki)
33	Seaweeds (e.g. hijiki, kombu)
34	Mayonnaise (including salad dressed with mayonnaise)
35	Deep fried food
36	Stir fried food
37	Mandarin, orange, grapefruit
38	Other fruits (e.g. strawbery, kiwi, apple, watermelon)
39	Peanuts, almond
40	Western confectioneries (e.g. cake, cream puff)
41	Japanese confectioneries (e.g. manju)
42	Green tea
43	Coffee
**Alcohol**	
1	Beer
2	Sake
3	Shochu
4	Whisky
5	Wine
6	Others (e.g. plum wine, chuhai)

B. Frequency and quantity

Staple food (asked at breakfast, lunch and dinner)	
Never or seldom	Number of portions per serving
1-3 times per month	Number of portions per serving
1-2 times per week	Number of portions per serving
3-4 times per week	Number of portions per serving
5-6 times per week	Number of portions per serving
Everyday	Number of portions per serving
**Food item**	**Weight**
Never or seldom	0.05
1-3 times per month	0.1
1-2 times per week	0.2
3-4 times per week	0.5
5-6 times per week	0.8
Once per day	1
Twice per day	2
Three or more times per day	3
**Alcohol**	
Never	-
Number of days per month	Number of drinks per occasion
Number of days per week	Number of drinks per occasion

The questionnaires were created based on the semi-quantitative food frequency questionnaire developed by the Department of Health Promotion and Preventive Medicine, Graduate School of Medicine, Nagoya City University.

Ref.) Tokudome S, Got C, Imaeda N, Tokudome Y, Ikeda M, Maki S. Development of a data-based short food frequency questionnaire for assessing nutrient intake by middle-aged Japanese. A*sian Pacific journal of cancer prevention* : *APJCP* . 2004; 5(1):40-3.
