# Supplementary material for: Charged metabolite biomarkers of food intake assessed via plasma metabolomics in a population-based observational study in Japan
Source: PLoS One. 2021 Feb 10;16(2):e0246456. doi: 10.1371/journal.pone.0246456 (PMC7875413; doi:10.1371/journal.pone.0246456)
Supplement: S2 Table — (PDF) [file pone.0246456.s005.pdf]

S2 Table. Instruments and analytical conditions

**Instruments**		
CE-TOF-MS	Agilent 7100 CE system	(Agilent Technologies, Waldbronn, Germany)
	Agilent 6224 LC/MS TOF system	(Agilent Technologies, Santa Clara, CA, USA)
	Agilent 1260 series isocratic HPLC pump	(Agilent Technologies, Santa Clara, CA, USA)
	G1603A Agilent CE-MS adapter kit	(Agilent Technologies, Santa Clara, CA, USA)
	G1607A Agilent CE-ESI-MS sprayer kit	(Agilent Technologies, Santa Clara, CA, USA)
Software	CE: Agilent Chemstation	(Agilent Technologies, Waldbronn, Germany)
	TOF-MS: Agilent MassHunter	(Agilent Technologies, Santa Clara, CA, USA)
	Raw data analysing: MasterHand	(proprietary software)
**Analytical conditions**		
Cationic mode		
	Column	Fused-silica capillary column (50 µm i.d. × 100 cm total length)
	Electrolyte	Formic acid (1 M)
	Sample injection	5 kPa for 3s (approximately 3 nL)
	Applied voltage	30 kV
	Sheath liquid	Methanol/water (50%v/v) containing 0.1 µM Hexakis
	Mass flow rate	10µL/min
	Reference standards	^{13}C isotopic ion of the protonated methanol dimer $(2MeOH + H)^+$, *m/z* 66.06306
		Protonated Hexakis $(M + H)^+$, *m/z* 622.02896
	Mass spectra	1.5 cycles/s from *m/z* 50 to 1000
Anionic mode		
	Column	COSMO(+) capillary column (Nacalai Tesque, Kyoto, Japan)
	Electrolyte	Ammonium acetate solution (50 mM, pH 8.5)
	Sample injection	5 kPa for 30 s (approximately 30 nL)
	Applied voltage	-30 kV
	Sheath liquid	Ammonium acetate (5 mM) in methanol/water (50 %v/v) containing 0.01 µM Hexakis
	Mass flow rate	10 µL/min
	Reference standards	^{13}C isotopic ion of the protonated methanol dimer $(2CH_3COOH - H)^-$, *m/z* 120.03834
		Hexakis + deprotonated acetic acid $(M + CH_3COOH - H)^-$, *m/z* 680.03554
	Mass spectra	1.5 cycles/s from *m/z* 50 to 1000

Ref.) Hirayama A, Sugimoto M, Suzuki A, Hatakeyama Y, Enomoto A, Harada S, et al. Effects of processing and storage conditions on charged metabolomic profiles in blood. *Electrophoresis*. 2015.
