# Supplementary material for: Charged metabolite biomarkers of food intake assessed via plasma metabolomics in a population-based observational study in Japan
Source: PLoS One. 2021 Feb 10;16(2):e0246456. doi: 10.1371/journal.pone.0246456 (PMC7875413; doi:10.1371/journal.pone.0246456)
Supplement: S3 Table — (PDF) [file pone.0246456.s006.pdf]

S3 Table. List of metabolites.

Cations	Sub class[a]	Anions	Sub class[a]
Glycine	AA	Pyruvate	AKA
Trimethylamine-*N*-oxide	AO	Lactate	AHA
beta-Alanine	AA	2-Oxobutyrate	SKA
Alanine	AA	Malonate	DCA
Sarcosine	AA	2-Hydroxybutyrate	AHA
3-Aminoisobutyrate	AA	3-Hydroxybutyrate	BHA
2-Aminobutyrate	AA	Fumarate	DCA
N,N-Dimethylglycine	AA	2-Oxoisopentanoate	SKA
Choline	QA	Hexanoate	FAE
Serine	AA	Succinate	DCA
Creatinine	AA	Isethionate	OSA
Proline	AA	5-Oxoproline	AA
Guanidinoacetate	AA	Citraconate	FA
Valine	AA	Ketoleicine	SKA
Betaine	AA	4-Acetylbutyrate	FA
Threonine	AA	Heptanoate	FA
Taurine	OSA	Glutarate	DCA
Pipecolate	AA	Malate	BHA
Hydroxyproline	AA	Threonate	CHO
Creatine	AA	Ethanolamine phosphate	PHE
Isoleucine	AA	Octanoate	FA
Leucine	AA	2-Oxoglutarate	GKA
Asparagine	AA	Pelargonate	FA
Ornithine	AA	Terephthalate	BA
Aspartate	AA	Urate	PR
Hypoxanthine	PR	Glycerophosphate	GP
1-Methylnicotinamide	PCA	Decanoate	FA
Trigonelline	AL	trans-Aconitate	TCA
Proline betaine	AA	cis-Aconitate	TCA
gamma-Butyrobetaine	FA	*N*-Acetylaspartate	AA
Glutamine	AA	Hippurate	BA
Lysine	AA	Homovanillate	MP
Glutamic acid	AA	Azelate	FA
Methionine	AA	Isocitrate	TCA
Triethanolamine	AM	Citrate	TCA
Histidine	AA	Quinate	ALC
alpha-Aminoadipate	AA	Glucuronate	CHO
Carnitine	QA	Cysteine-*S*-sulfate	AA
Phenylalanine	AA	Galactarate	CHO
3-Methylhistidine	AA	3-Indoxyl sulfate	ARX
Arginine	AA		
Guanidinosuccinate	AA		
Indole-3-acetate	ICA		
Citrulline	AA		
Tyrosine	AA		
Symmetric Dimethylarginine	AA		
Asymmetric Dimethylarginine	AA		
o-Acetylcarnitine	LP		
Tryptophan	AA		
Kynurenine	CAB		
Cysteineglutathione disulfide	AA		
Cystine	AA		
Uridine	PYN		
Glycerophosphorylcholine	GPC		

[a] Ref.) The Human Metabolome Database (http://www.hmdb.ca)

Abbreviations	
AA	Amino acids, peptides, and analogs
AHA	alpha-Hydroxy acids and derivatives
AKA	alpha-Keto acids and derivatives
ALK	Alkaloids and derivatives
ALC	Alcohols and polyols
AM	Amines
AO	Aminoxides
ARX	Arylsulfates
BA	Benzoic acids and derivatives
BHA	beta-Hydroxy acids and derivatives
CHO	Carbohydrates and carbohydrate conjugates
CAB	Carbonyl compounds
DCA	Dicarboxylic acids and derivatives
FA	Fatty acids and conjugates
FAE	Fatty acid esters
GKA	gamma-Keto acids and derivatives
GP	Glycerophosphates
GPC	Glycerophosphocholines
ICA	Indolyl carboxylic acids and derivatives
LP	Lipids and lipid-like molecules
MP	Methoxyphenols
OSA	Organosulfonic acids and derivatives
PCA	Pyridinecarboxylic acids and derivatives
PHE	Phosphate esters
PYN	Pyrimidine nucleosides
PR	Purines and purine derivatives
QA	Quaternary ammonium salts
SKA	Short-chain keto acids and derivatives
TCA	Tricarboxylic acids and derivatives
