# Supplementary material for: Charged metabolite biomarkers of food intake assessed via plasma metabolomics in a population-based observational study in Japan
Source: PLoS One. 2021 Feb 10;16(2):e0246456. doi: 10.1371/journal.pone.0246456 (PMC7875413; doi:10.1371/journal.pone.0246456)
Supplement: S4 Table — (PDF) [file pone.0246456.s007.pdf]

S4 Table. Summary of cross-validation analyses

Food group	Minimum mean PRESS	Minimaizing number of factors	Optimal number of factors[a]	Cumulative R^2X [b]	Cumulative R^2Y [c]	Cumulative Q^2 [d]
Meat	0.97	4	3	0.20	0.05	0.07
Fish/seafood	0.91	4	3	0.21	0.10	0.21
Eggs	0.99	4	1 (2)	0.11 (0.14)	0.01 (0.03)	0.01 (0.02)
Dairy	0.89	11	4	0.24	0.13	0.33
Soy products	0.92	10	4	0.24	0.10	0.23
Carotenoide-rich vegetables	0.88	4	3	0.21	0.13	0.28
Other vegetables	0.87	10	3	0.21	0.14	0.31
Fruits	0.83	5	4	0.24	0.19	0.47
Green tea	0.97	3	2	0.11	0.04	0.05
Coffee	0.79	12	4	0.24	0.23	0.55
Alcohol[e]	0.74	10	3	0.14	0.29	0.53

PRESS, predicted residual sum of squares.

[a] Smallest number of factor numbers provided by Van der Voet test with a T^2 critical value of $p > 0.10$. For cases with an optima factor number of less than two, the factor number was set to two and the result was shown in parentheses.

[b] Cumulative explained variation in the X matrix.

[c] Cumulative explained variation in the Y matrix.

[d] Cumulative predicted variation in the Y matrix.

[e] Data of male drinkers were used in the analysis.
